# Astrocytes in Genetic Epilepsies: Supporting Actor or Key Player?

**DOI:** 10.1002/jnr.70148

**Published:** 2026-08-06

**Authors:** Jenny Lange, Erica Zhao, Ellie O'Connell, Olivia Gillham, Amy McTague

**Affiliations:** ^1^ UCL UK Dementia Research Institute London UK; ^2^ Department of Physiology, Anatomy and Genetics, Institute of Developmental and Regenerative Medicine University of Oxford Oxford UK; ^3^ Developmental Neurosciences, Zayed Centre for Research Into Rare Disease in Children UCL Great Ormond Street Institute of Child Health London UK; ^4^ Department of Cell and Developmental Biology University College London London UK

**Keywords:** astrocytes, epilepsy, neurodevelopment

## Abstract

Epilepsy is one of the most common neurological disorders worldwide, affecting around 1% of the population. The epilepsies represent a diverse group of conditions, ranging from acquired forms resulting from neurological insults to common multifactorial epilepsies and rare, often monogenic epilepsies caused by highly penetrant genetic variants. The genetic epilepsies demonstrate frequent comorbidity with a range of neurodevelopmental and psychiatric disorders, and epileptic seizures are also a common feature of neurodevelopmental disorders such as Fragile X syndrome and Rett syndrome. Astrocytes, the most numerous glial cells in the central nervous system, have emerged as crucial players in the pathophysiology of acquired epilepsies. Whilst the contribution of astrocytes to acquired epilepsy has been widely reviewed, astrocyte dysfunction in rare genetic epilepsies or neurodevelopmental disorders has been neglected, despite the fact that the genes implicated are expressed in astrocytes, albeit to a lesser extent than in neurons. Additionally, affected individuals with rare genetic epilepsies are more likely to exhibit drug‐resistant seizures, highlighting the need to identify novel therapeutic targets. In this paper, we review the existing literature on astrocyte dysfunction in genetic epilepsy syndromes and neurodevelopmental disorders with seizures. We have identified several key studies that highlight alterations in crucial astrocyte functions including calcium signaling and ion homeostasis. Our review highlights the need for further research to establish the contribution of astrocyte dysfunction to neuronal health and seizure activity in rare genetic epilepsies.

## Significance Statement

The role of astrocytes in acquired epilepsy has been extensively studied and reviewed, with evidence that astrocyte can contribute to and worsen epileptiform activity and seizure propagation. However, their role in genetic epilepsies and their contribution to seizure activity in neurodevelopmental disorders has received less attention. This review summarizes current knowledge of astrocytic phenotypes in genetic epilepsy syndromes and explores whether these are cell‐intrinsic or responses to neuronal seizure activity. Elucidating the mechanisms of astrocyte dysfunction in these disorders is not only crucial for understanding pathology but also for the development of novel therapies that target all affected cell types.

## Graphical abstract

Astrocytes contribute to the pathophysiology of acquired epilepsy. However, less is known about their contribution to genetic epilepsy syndromes which often exhibit frequent comorbidity with neurodevelopmental and psychiatric disorders. Epileptic seizures are also frequently present in neurodevelopmental disorders. Here, we review what is known about changes in astrocyte functions in genetic epilepsy syndromes and neurodevelopmental disorders that feature epileptic seizures.

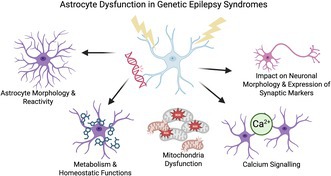

Epilepsy is a chronic neurological disorder characterized by recurrent unprovoked seizures, affecting 0.5%–1% of the worldwide population (Milligan 2021; Tellez‐Zenteno et al. 2007). Patients with epilepsy have an increased risk of co‐morbid disorders and increased premature mortality. Despite the development of new antiseizure medications (ASMs), one third of affected individuals continue to experience seizures.

The epilepsies represent a range of conditions including common and rare epilepsies, with the former largely caused by polygenic genetic influences and/or environmental factors, and the latter by pathogenic variants affecting a number of cellular pathways (Van Loo et al. 2022). More than 900 genes have been associated with epilepsy to date, with more than 90% of genetic epilepsies associated with developmental delay or regression, also known as developmental and epileptic encephalopathies (Oliver et al. 2023; Scheffer et al. 2024). Epilepsy can also occur secondary to a neurological insult, such as traumatic or hypoxic brain injury, brain tumors, central nervous system infections, stroke and status epilepticus (SE), resulting in acquired or symptomatic epilepsies (AE). The initial insult is followed by a latent and then chronic phase with recurrent spontaneous seizure activity (Fisher et al. 2005). Temporal lobe epilepsy (TLE), particularly mesial temporal lobe epilepsy (MTLE), is a common form of acquired epilepsy (Bertram 2009; Heinemann et al. 2000). TLE is often drug resistant and can be triggered by insults including hypoxia, infections or tumors, with complex febrile seizures also being a risk factor (Engel Jr. 2001; Fisher et al. 2005).

The process which transforms the healthy brain into an epileptic brain with a propensity for spontaneous seizures is referred to as epileptogenesis and is accompanied by functional and structural alterations (Pitkänen and Lukasiuk 2011). Alongside neuronal subpopulations, glial cells including astrocytes have been implicated in epileptogenesis. Astrocytes are the most numerous glial cells in the human central nervous system (CNS) and are key players in many crucial brain functions such as learning and memory (Khakh and Sofroniew 2015; Sofroniew and Vinters 2010). Traditionally, astrocytes were thought to be a homogeneous cell population, but recent research has highlighted a remarkable heterogeneity in astrocyte development and function (Holt 2023). Furthermore, it has been suggested that astrocytes may be matched to neuronal subpopulations to support local circuits. Astrocytic processes ensheath synapses, thus making them part of the synaptic structure alongside pre‐ and postsynaptic elements, known as the tripartite synapse (Semyanov and Verkhratsky 2021) where they can modulate neuronal activity. They also form part of the innate immune response of the brain, where they become reactive in response to injury and disease (Escartin et al. 2021) and undergo morphological, molecular and functional changes.

The role of astrocytes in AE represents an extensive field of study that has been reviewed in detail (Devinsky et al. 2013; Purnell et al. 2023; Sumadewi et al. 2023; Vezzani et al. 2022). Here, we briefly review the potential mechanisms and contribution of astrocyte dysfunction to AE (Figure 1) to provide context for their possible role in genetic epilepsies and neurodevelopmental disorders where epileptic seizures represent a major clinical phenotype. In this narrative review, we focus on research investigating astrocyte phenotypes in genetic epilepsies (Table 1) or neurodevelopmental disorders with an epileptic component (Table 2). Research papers were included on the basis that astrocyte function was investigated in models carrying known pathogenic variants. Neuronal mechanisms or contributions of other glial cells were considered beyond the scope of this review.

**FIGURE 1:**
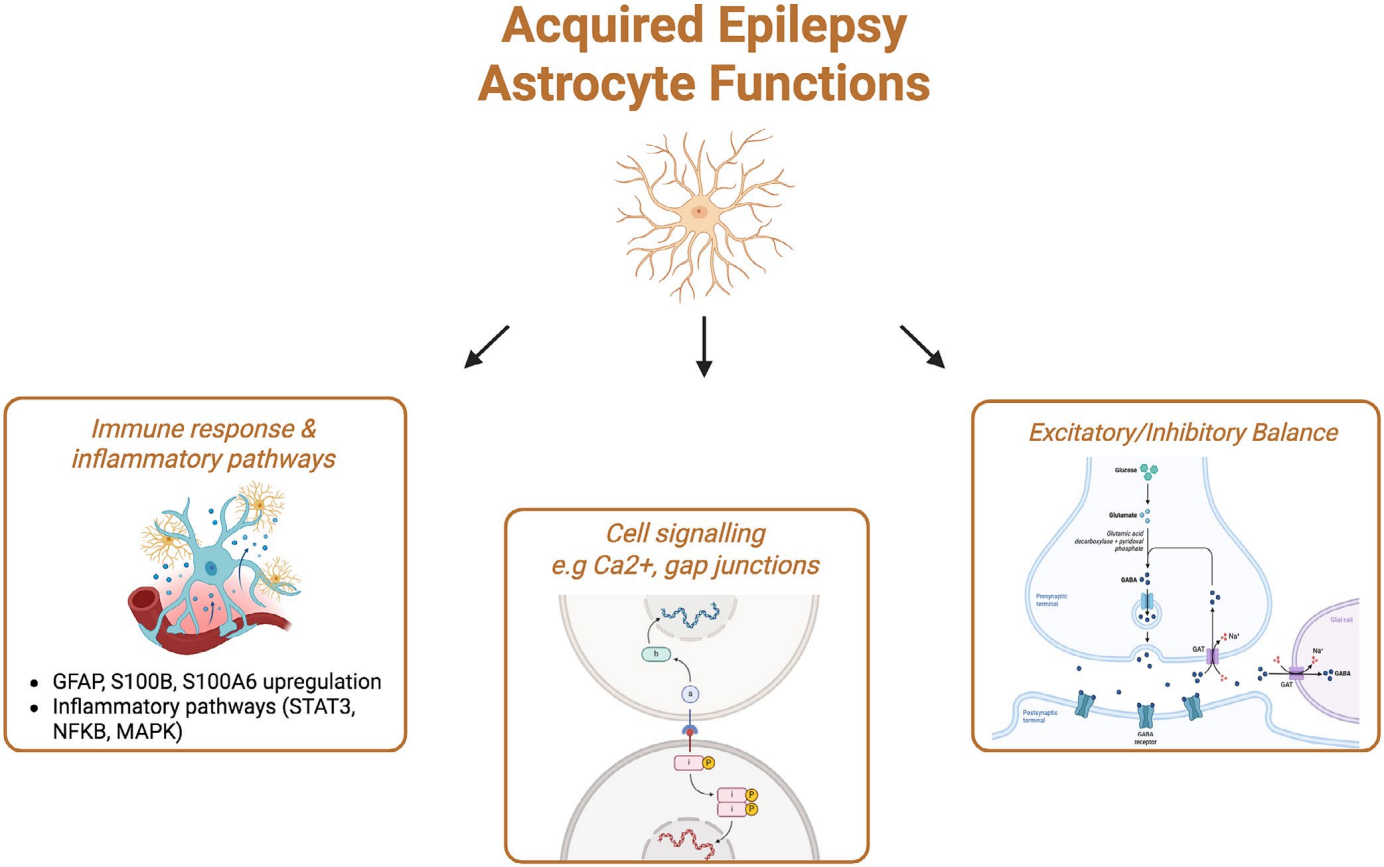
Astrocyte phenotypes in acquired epilepsy.

**TABLE 1:** Astrocyte phenotypes in genetic epilepsies.

Paper	Gene/disorder	Primary astrocyte phenotype	Astrocyte phenotype: reactive or cell intrinsic?	Seizure phenotype	Model	Age of animal model	Time course between experimental evaluation and seizure onset	Presence of SE in animal model	Therapeutic implications
Kim et al. (2021) (as above)	*KCNQ2*/DEE	Increased number of astrocytes widespread upregulation of GFAP	Reactive	Spontaneous seizures, KA‐induced	Conditional transgenic mouse model carrying a KCNQ2 patient variant	P120–P180	KA induced: recordings for 120 min	Not defined	N/A
Sicca et al. (2016)	*KCNJ10*/EAST Syndrome/SeSAME syndrome	Whole cell current densities were significantly larger in mutant cells enhancing K^+^ influx Mislocalisation of Kir4.1	Intrinsic	No	*Xenopus laevis* oocytes, astrocytoma cell lines expressing WT or mutant Kir4.1 channels	N/A	N/A	N/A	N/A
Djukic et al. (2007)	*KCNJ10*/EAST Syndrome/SeSAME syndrome	Increase in membrane resistance, membrane depolarization Impaired glutamate uptake capacity	Intrinsic	Spontaneous seizures in mouse model	Astrocyte conditional mouse knock‐out (cKO) of Kir4.1	P15–P20	N/A	Yes	N/A
Mermer et al. (2022)	*SLC6A1/GAT1*	Reduced GABA uptake Reduced GAT‐1 expression I GAT‐1 re‐localisation to ER	Intrinsic	Absence seizures in mouse model, but astrocyte phenotypes established in culture	Primary mouse astrocytes transfected with a patient variant, patient iPSC derived astrocytes	N/A	N/A	N/A	N/A
Poliquin et al. (2021)	*SLC6A1/GAT1*	Reduced GABA uptake	Intrinsic	No	Primary mouse astrocytes transfected with a patient variant	N/A	N/A	N/A	N/A
Nwosu et al. (2022)	*SLC6A1/GAT1*	Reduced GABA uptake GAT‐1 retention to ER	Intrinsic	Not in the model, but *SLC6A1* ^*+/S295L*^ knockin mice have seizures	Primary astrocytes isolated from SLC6A1 knock in mice, patient iPSC derived astrocytes	2–4 months	N/A	Yes	4Phenylbutyrate restored GABA uptake capacity in astrocytes and increased GAT‐1 expression, reduced seizures In mice
Mermer et al. (2021)	*SLC6A1/GAT1*	Reduced GABA uptake GAT‐1 mislocalisation to ER	Intrinsic	No	Patient iPSC derived astrocytes	N/A	N/A	N/A	N/A
Henke et al. (2020)	*SLC13A5*/DEE	Increased citrate in and decreased lactate levels in the CSF of mice	Unclear	Spontaneous seizures, PTZ induced seizures	SLC13A5^−/−^ mice	> 7 weeks	N/A	N/A	N/A
Salazar et al. (2023)	*SCN1A*/Dravet Syndrome	Reactive astrocytes in retinal tissue	Reactive	Model has spontaneous seizures, but not reported here	DS knock in mouse Syn‐Cre/Scn1a^WT/A1783V^ (exclusive expression in neurons in CNS)	25 days	N/A	N/A	N/A
Martín‐suárez et al. (2020)	*SCN1A*/Dravet Syndrome	Increased GFAP intensity increased number of GFAP+/S100b + astrocytes Increased morphological complexity	Reactive	Model has spontaneous seizures, but not reported here	DS knock in mouse Syn‐Cre/Scn1a^WT/A1783V^ (exclusive expression in neurons in CNS)	7 week old	N/A	N/A	N/A
Valassina et al. (2022)	*SCN1A*/Dravet Syndrome	Increased morphological complexity Transcriptomic changes associated with astrocyte reactivity	Reactive	Yes, following body temperature increase to mimic febrile seizures	*Scn1a* ^Stop/+^ mouse model	P30 onwards Seizure measurement D45–55	N/A	Not defined	SCN1A re‐expression rescues seizure phenotype, astrocytes not further assessed
Goisis et al. (2022)	*SCN1A*/Dravet Syndrome	Increased GFAP expression in entorhinal cortex and dentate gyrus	Prior to seizure onset	Yes, spontaneous seizures	Heterozygous (Na_V_1.1^+/−^; DS mice)	P15–19 for pre‐seizure, P22 for post seizures	N/A	Not defined	N/A
Satta et al. (2021)	*SCN1A*/Dravet Syndrome	Increased astrocyte proliferation	Reactive	Yes, spontaneous convulsions and greater susceptibility to PTZ induced seizures	Syn‐Cre/Scn1a^WT/A1783V^ (exclusive expression in neurons in CNS)	D25	Measured 30 min post injection	Not defined	N//A
Tiraboschi et al. (2020)	*SCN1A*/Dravet Syndrome	Reduced astrocyte maturation, increased astrocyte proliferation	Unclear	Yes, spontaneous seizures	Zebrafish scn1lab^mut/mut^	4–7 day postfertilization larvae	20 min recordings	Not defined	Fenfluramine treatment rescued neuronal deficits, astrocytosis not assessed
Uchino et al. (2023)	*SCN1A*/Dravet Syndrome	Ca^2+^ signaling was altered, with an increased slope of spontaneous and ATP‐induced Ca^2+^ spiking	Intrinsic	No, astrocytes were stimulated with ATP (1 μM)	Mouse with a loss of function pathogenic variant in *SCN1A* *Scn1a* ^+/−^ mice	P0–P1 primary astrocyte cultures	N/A	N/A	N/A
Cherkezzade et al. (2024)	*ALG13*/Lennox Gastaut	Elevated levels of YKL‐40	Reactive	Yes	Human Serum	N/A	N/A	N/A	N/A
Gao et al. (2019)	*ALG13*/Lennox Gastaut	Increased number of GFAP positive astrocyte, astrocyte hypertrophy and glial scar formation around areas of neuronal loss	Reactive	Yes, KA injection & pilocarpine‐induced epilepsy model	*ALG13* ^*−/−*^ mice	12 week old mice	Recorded for 60/120 min, diazepam to weaken SE Culled 1 h, 4 h, 8 h, 24 h, and 7 d,14 d, and 21 d post SE	Yes	N/A
Hussain et al. (2019)	*WWOX*‐related DEE	Evidence of astrogliosis upregulation of cytokines TNF‐a and IL‐6	Unclear	No seizures	Wwox^−/−^ mice	2 weeks old	N/A	N/A	N/A
Hussain et al. (2023)	*WWOX*‐related DEE Spinocerebellar ataxia with epilepsy and intellectual disability (SCAR12)	Astrocyte reactivity, upregulation of pro‐inflammatory pathways	Unclear, as age of seizure onset not reported	Spontaneous seizures	WWOX‐loss of function WwoxP47T/P47T	Age not listed	Recorded over 21‐53 h	Generalized tonic–clonic convulsive episodes	N/A
Steinberg et al. (2021)	*WWOX*‐related DEE	Increased number of GFAP and S100b positive astrocytes, upregulation of astrocyte markers	Unclear	No, organoid model, but evidence of neuronal hyperexcitability and epileptiform activity following 100 μM 4‐AP	Human iPSC‐derived organoids WWOX Ko and patient derived (c.517‐2A>G splice site mutation)	7 weeks	Recording for 30 min	N/A	Phenotypes reversed by restoring WWOX
Repudi et al. (2021)	*WWOX*‐related DEE	No astrocyte changes reported	N/A	Neocortical hyperexcitability, in vitro and in vivo, large amplitude bursting activity	Conditional WWOX Ko mice (*Wwox* ^*flox*/*flox*^; *Nestin‐Cre* ^+^), S‐KO (*Wwox* ^*flox*/*flox*^; *Synapsin‐Cre* ^+^), O‐KO (*Wwox* ^*flox*/*flox*^; *Olig2* ^*cre/+*^) and G‐KO (*Wwox* ^*flox*/*flox*^; *Gfap‐Cre* ^+^)	Age not listed	Recorded for 20 min in vivo	Not defined	N/A
Sprissler et al. (2018)	*SCN8A*‐related DEE	Upregulation of GFAP in hippocampal astrocytes Upregulation of markers associated with astrocyte reactivity (RNA seq)	Reactive, pre‐seizure vs. post seizure	Yes	D/+ mice (patient missense pathogenic variant) *Scn8a* ^N1768D^ knock‐in mice	6 weeks pre seizure, 14 weeks post seizure	0.5 to 3 min with onset	Not defined	N/A
Thompson et al. (2022)	*SCN8A*‐related DEE	Number of GFAP+ astrocytes increased in both cortex and hippocampus, downregulation of GS Altered Kir4.1 signaling	Reactive, Few GFAP positive astrocytes pre‐seizure age (P21‐P24)	Yes, spontaneous seizures	D/+ mice (patient missense pathogenic variant) *Scn8a* ^N1768D^ knock‐in mice	8 and 16 weeks postnatal.	Recorded over 2–4 days	Not defined	N/A
Marin‐valencia et al. (2012)	Glucose transporter 1 (Glut‐1) deficiency syndrome (G1D) *SLC2A1*	Enhanced GFAP reactivity at D28 and D120 (more pronounced with age)	Pre‐seizure, but likely reactive	Manifested generalized jerks at rest and upon walking G1D mice had frequent epileptic spikes and series of spike and spike–wave activity	G1D mice	Between 3 and 5 months of age e	Repetitive epileptiform spike and spike wave activity generally persisted for 3–10 s and was associated with an increase of power in the theta frequency band (5–8 Hz) at each event 4–8 events per hour	Not defined	N/A
Kim et al. (2013)	Glucose transporter 1 (Glut‐1) deficiency syndrome (G1D) *SLC2A1*	Increased glucose uptake	Intrinsic	No	Primary astrocyte cultures prepared from heterozygous SLC2A1 mice	N/A	N/A	N/A	Valproate treatment increased glucose uptake in astrocytes (48 h treatment)
Ullner et al. (2009)	Glucose transporter 1 (Glut‐1) deficiency syndrome (G1D) *SLC2A1*	Astrogliosis	Unclear	No	G1D mice Glut‐1^+/−^ mouse model	P21	N/A	N/A	N/A
Goubert et al. (2017)	*SLC25A22*	Reduced nicotinamide adenine dinucleotide (Phosphate) (NAD(P)H) formation after glutamate stimulation, cytosolic glutamate accumulation	Intrinsic, following glutamate stimulation	No	Primary astrocyte monocultures (siRNA silencing)	N/A	N/A	N/A	N/A
Chan et al. (2019)	POLG	Astrogliosis in brain tissue and acute slices	Reactive	Yes	Patient tissue	N/A	N/A	N/A	N/A
Smith et al. (2023)	Alper's syndrome (bi‐allelic *POLG* mutations)	Hypertrophic, GFAP‐reactive astrocytes Reduced mitochondrial complex I subunit (NDUFB8) and complex IV subunit (COXI) in GFAP positive astrocytes Increased Kir4.1 & AQP4 levels, decreased GS in 4/6 patients	Unclear	Refractory epilepsy was the main presenting symptom in all patients	Patient tissue	N/A	N/A	Not defined	N/A
Craciun et al. (2025)	*TCF4*	Astrocyte reactivity 60% reduction of TCF4 expression in astrocytes (30% in other cell types including neurons)	Unclear, alongside increased neuronal activity Astrogliosis present from Day 4, seizures from 3 months of age	Yes	TCF4 heterozygous deletion Mice	3+ months of age	Seizures recorded when placed in cage, recording for 10 min	Not defined	N/A

Abbreviation: N/A, not applicable.

**TABLE 2:** Astrocyte phenotypes in neurodevelopmental disorders.

Paper	Disorder/gene	Primary astrocyte phenotype	Astrocyte phenotype: reactive or cell intrinsic?	Seizure phenotype	Model	Age of animal model	Time course between experimental evaluation and seizure onset	Presence of SE in animal model	Therapeutic implications
Jacobs and Doering (2010)	Fragile X syndrome (FXS) (FMRI)	Not reported	Intrinsic, effect on WT neurons	No, hyperexcitability not assessed	Astrocyte/Neuron co‐cultures *Fmr1* KO mice	N/A	N/A	N/A	N/A
Ren et al. (2023)	Fragile X syndrome (FXS) *FMRI*	Increased astrocyte number (postmortem tissue) Enhanced Ca^2+^ signaling in response to ATP application Altered metabolism/Cholesterol metabolism (iPSC‐derived astrocytes)	Intrinsic, astrocyte only cultures	No	Astrocytes derived from FXS patient iPSCs and *FMR1* ^−/−^ knock out hESCs	N/A	N/A	N/A	N/A
Reynolds, Napier, et al. (2024)	Fragile X syndrome (FXS) *FMRI*	Defects in purinergic signaling	Intrinsic	No	Primary *FMR1* ^−/−^ astrocytes *Fmr1* KO mouse *Fmr1* KO; FVB.129P2(B6)‐Fmr1^tm1Cgr^	N/A	N/A	N/A	N/A
Reynolds et al. (2021)	Fragile X syndrome (FXS) *FMRI*	Upregulation of purinergic receptors in *FMR1* ^−/−^ astrocytes had previously been reported, whilst application of ATP and UTP evoked greater Ca^2+^ responses than in WT astrocytes, suggesting a role for purinergic signaling in aberrant *FMR1* ^−/−^ astrocyte activation	Intrinsic	No	primary *FMR1* ^−/−^ astrocytes *Fmr1* KO mouse *Fmr1* KO; FVB.129P2(B6)‐Fmr1^tm1Cgr^	N/A	N/A	N/A	N/A
Reynolds, Huang, et al. (2024)	Fragile X syndrome (FXS) *FMRI*	Not reported, impact on neuronal health	N/A	No, but network activity assessed by MEA, reduced firing synchronicity of neurons	Primary *FMR1* ^−/−^ astrocytes *Fmr1* KO mouse *Fmr1* KO; FVB.129P2(B6)‐Fmr1^tm1Cgr^	N/A	N/A	N/A	N/A
Jin et al. (2021)	Fragile X syndrome (FXS) *FMRI*	Astrocyte phenotypes not reported, but specific loss of FMRI in astrocytes resulted in cortical hyperactivity and behavioral abnormalities	Intrinsic	No seizures, but changes in neuronal excitability	*FMR1* ^−/−^ knockout mouse model (conditional astrocyte KO model)	Juvenile (P24–P30) and young adult (P38–P45)	N/A	N/A	N/A
Sharma et al. (2023)	Fragile X syndrome (FXS) *FMRI*	Astrocyte phenotypes not reported, but FMRI astrocytes increased neuronal excitability	N/A	N/A	iPSC‐derived astrocytes CS848iFXS‐n5 CS072iFXS‐n4 Shef 4‐FMR1 null	N/A	N/A	N/A	Addition of S100b to FXS astrocytes corrected aberrant neuronal firing
Bataveljic et al. (2024)	Fragile X syndrome (FXS) *FMRI*	Activity dependent increase in extracellular K^+^ levels in the hippocampus *FMR1* ^−/−^ astrocytes exhibited reduced peak amplitude of Kir4.1 currents Reduced Kir4.1 expression in hippocampal *FMR1* ^−/−^ astrocytes	Unclear	Enhanced neuronal excitability in acute hippocampal slices	*FMR1* ^−/−^ knockout mouse model	P21‐30	N/A	N/A	Viral delivery of Kir4.1 rescues activity‐dependent K^+^ uptake in FMRP‐deficient astrocytes Viral delivery of Kir4.1 in astrocytes normalizes neuronal excitability in *Fmr1* knockout mic
Pejhan et al. (2020)	Rett Syndrome *MECP2*	Lower MECP2 expression in astrocytes from patients vs. healthy controls	Unclear	No seizures or patient phenotypes reported	*Patient post mortem tissue*	Patient ages: 13, 19, 20, 44 Age matched controls	N/A	N/A	N/A
Segatto et al. (2014, 2019)	Rett Syndrome *MECP2*	Elevated cholesterol levels	Unclear	Not reported	*Patient serum*	Not reported	N/A	N/A	N/A
Sun et al. (2023)	Rett Syndrome *MECP2*	Widespread transcriptional changes, impaired metabolic homeostatic activity Altered astrocyte morphology	Intrinsic for astrocyte mono cultures, cell autonomous and non‐cell autonomous in co‐cultures	No	hPSC‐derived astrocytes with *MECP2* pathogenic variants R270X (C808T) and R133C (C397T) mutations via CRISPR/Cas9 technology^*23*^	N/A	N/A	N/A	N/A
Bebensee et al. (2017)	Rett Syndrome *MECP2*	Altered mitochondrial metabolism	Intrinsic	No, astrocytes only cultures	MeCP2‐deficient mouse (*Mecp2* ^*−/y*^) Cultured astrocytes	2‐ to 4‐day‐old mice	N/A	N/A	N/A
Tomasello et al. (2024)	Rett Syndrome *MECP2*	Mitochondrial abnormalities	Organoids – not clear if intrinsic due to model h‐ESC derived astrocytes & metabolism: intrinsic	No network activity reported, but changes in expression of excitatory‐inhibitory balance markers (increase in excitatory neurons)	hESC‐derived organoids, WIBR3 and WIBR1lines with isogenic controls	Organoids: D100	N/A	N/A	N/A
Kahanovitch et al. (2018)	Rett Syndrome *MECP2*	Reduced Kir4.1 mRNA and protein in Mecp2^−/−^ astrocytes, changes in Kir4.1 mediated currents	Kir4.1 currents: unclear as not single astrocyte cultures, MeCP2 regulation of Kir4.1 astrocytes cell autonomous as astrocyte monocultures	No seizures reported	Mecp2^−/−^ mice	P50, 7–8 months of age	N/A	N/A	N/A
Okabe et al. (2012)	Rett Syndrome *MECP2*	Abnormal glutamate clearance changes in EAAT1/EAAT2 expression Increased levels of glutathione protein	Intrinsic in monocultures	No seizures reported in mice	Mecp2^−/−^ mice *Astrocyte cultures*	P0‐P1 mice for astrocyte cultures	N/A	N/A	N/A
Maezawa et al. (2009)	Rett Syndrome *MECP2*	Reduced MECP2 expression facilitated trough gap junctions	Intrinsic Non‐cell autonomous spreading of MeCP2	No	*Cultures* WT astrocytes co‐cultured with mosaic MeCP2^+/−^astrocytes (*Mecp2* ^*tm1.1Bird/+*^ *mic*)	P1 mice for cultures, experiments at 2–4 weeks in vitro	N/A	N/A	N/A
Dong et al. (2018)	Rett Syndrome *MECP2*	Abnormal Ca^2+^ activity in astrocytes	Ca2^+^ in astrocyte monocultures, intrinsic Changes observed in astrocyte specific knockdown but not neuron specific	No, but increased neuronal network activity in acute slices	Mecp2^−/−^ mice (R294X‐MT) Primary astrocytes Acute brain slices RTT iPSC lines (R294X mutation)	Epileptiform activity in acute slices from 5 to 8 weeks mice	N/A	N/A	Exogenous expression of MeCP2 rescues the abnormal calcium activities in RTT astrocytes.
Dong et al. (2020)	Rett Syndrome *MECP2*	Increases in GAT3 levels increased GAT3 mediated currents in hippocampal astrocytes	Intrinsic, Reduced tonic inhibition in astrocytes‐specific *Mecp2* KO mice	Epileptiform activity in acute slices	Astrocyte specific deletion Mice, full Knockout mice (*Mecp2* ^tm1.1jae^) (all cells) Acute slices	3–4 weeks old, 8–10 weeks old	20 min	N/A	N/A
Rakela et al. (2018)	Rett Syndrome *MECP2*	Reduced synaptic response to astrocytic stimulation compared to WT mice Reduced astrocytic Ca2^+^ signaling	Unclear	Not reported	MECP2 null mice, mosaic MECP2 mice (50% of cells express MECP2)	N/A	N/A	N/A	N/A
Albizzati et al. (2024)	Rett Syndrome *MECP2*	Transcriptomic analysis of Mecp2^−/−^ astrocytes revealed differential expression of genes associated with synapse maturation, whilst inflammatory pathways were upregulated in WT neurons co‐cultured with Mecp2^−/−^ astrocytes, which appeared to be driven by increased expression of IL‐6 by astrocytes.	Co‐cultures, unclear Increased IL‐6 from astrocytes appears to depend on neurons	No	Primary WT neurons co‐cultured with Mecp2^−/−^ astrocytes	Primary cultures from E15.5 mouse embryos, astrocytes from P1‐3	N/A	N/A	Inhibition of IL‐6 rescued pre‐synaptic puncta formation, but only partially restored post‐synaptic puncta
Hope et al. (2017)	Duplication 15q syndrome	Reduced K^+^ levels in glial cells	Unclear	Yes (mechanical stress “Bang” seizures)	Drosophila Overexpression of fly homolog in glia only	Measured bang‐sensitivity at 0–2 days, 3–6 days, and 7–10 days post‐eclosion	120 s	N/A	N/A

Abbreviation: N/A, not applicable.

## 1 Astrocyte Reactivity and Inflammation in Acquired Epilepsy

Astrocyte reactivity involves many pathways that may be disease dependent and are often not clearly defined. Furthermore, reactive astrocytes cannot be neatly divided into binary and outdated A1 “neurotoxic” or A2 “neuroprotective” categories (Escartin et al. 2021), and astrocytes in a number of diseases exhibit signatures from both categories (Takahashi et al. 2022). However, in a number of neurological diseases, it has become increasingly clear that astrocyte dysfunction significantly contributes to disease pathology (Pekny et al. 2016). In many different forms of epilepsy, including neurodevelopmental disorders with epilepsy, altered astrocyte functions may contribute to ictogenesis, the process leading to seizure activity, and epileptogenesis. The majority of insights into astrocyte dysfunction in epilepsy have emerged from models of acquired epilepsy (Binder and Steinhäuser 2021).

Upregulation of glial fibrillary acidic protein (GFAP) is seen as the first indicator of astrocyte reactivity and has been observed in a number of epilepsy models (Amhaoul et al. 2015; Chen et al. 2019; Yang et al. 2009) as well as in human brain tissue (Boer et al. 2010; Lu et al. 2019; Shapiro et al. 2008). Ca^2+^ binding proteins S100b and S100A6 are highly expressed by astrocytes and upregulated in reactive astrocytes, and have also been shown to be upregulated in astrocytes from in murine models, human post mortem tissue and patient serum (Maiti et al. 2018; Shapiro et al. 2008; Somera‐molina et al. 2007; Vizuete et al. 2018; Yamada and Jinno 2012; Yang et al. 2009) More recently, a new subtype of reactive astrocytes defined by elevated APOE expression and excessive lipid accumulation has been described in mouse models and human post mortem tissue from patients with TLE, that appear to contribute to disease progression (Chen et al. 2023). Increased proliferation of reactive astrocytes is seen, as is the case in several AE epilepsy models (Aronica et al. 2001; Tooyama et al. 2002; Verde et al. 2021; Vessal et al. 2004, 2005; Zhou et al. 2023). Several pro‐inflammatory pathways have been implicated in AE, including activation of the STAT3 pathway (Choi et al. 2003; Liang et al. 2023; Song et al. 2021; Xu et al. 2011) NF‐kB signaling (Crespel et al. 2002; Lerner‐natoli et al. 2000; Liu, Zhao, et al. 2023; Voutsinos‐porche et al. 2004) and MAPK pathways (Choi et al. 2003; Kim and Kang 2018) in astrocytes specifically.

Astrocytes, alongside microglia, are thought to contribute to inflammation in the epileptic brain through the release of pro‐inflammatory cytokines (Choi et al. 2009; Das et al. 2012; Kan, Van Der Hel, et al. 2012; Kan, De Jager, et al. 2012; Pernot et al. 2011), interleukins (Brain et al. 2012; Ravizza et al. 2008; Voutsinos‐porche et al. 2004; Xiong et al. 2016; Zhang et al. 2021) and to express proteins involved in pro‐inflammatory pathways (Zhang et al. 2021) including vascular endothelial growth factor (VEGF) (Cho et al. 2019; Sun et al. 2016; Twible et al. 2021) and complement component C3 (Li et al. 2022; Lin et al. 2021).

A number of ASMs have been shown to reduce astrocyte reactivity (Dariani and Baluchnejadmojarad 2013; Xia et al. 2018), whilst genetic induction of chronic astrogliosis has been shown to be sufficient in mice to induce epileptiform activity (Robel et al. 2009, 2015). Indeed, Robel et al. (2015) found that interictal spiking frequencies increased with age and correlated with worsening of astrocyte phenotypes, suggesting that seizures contribute to gliosis which in turn may exacerbate seizure phenotypes. In AE, astrocyte reactivity may be a consequence of brain injury or SE, rather than the primary cause of seizure activity. However, it is possible that continued astrocyte reactivity may contribute to epileptogenesis and aggravate neuronal seizure activity (Zhang et al. 2021). For example, inducing astrocyte reactivity led to network hyperactivity and reduced synaptic inhibition (Ortinski et al. 2010), suggesting that reactive astrocytes could contribute further to the excitation: inhibition imbalance and drive seizure activity.

## 2 Altered Cell Signaling in Acquired Epilepsy

Altered Ca^2+^ signaling in astrocytes is a commonly identified disease phenotype in neurological disorders, including neurogenerative diseases and neurodevelopmental disorders (Nedergaard et al. 2010). In AE, changes in Ca^2+^ signaling are thought to contribute to abnormal neuronal activity, ictogenesis and seizure generation. Heuser et al. (2018) reported in a kainate acid (KA) induced seizure mouse model that pronounced Ca^2+^ transients in astrocytes occur prior to local hypersynchronous neuronal activity, suggesting a role for astrocytes in early ictogenesis. Conversely, other studies found astrocytic Ca^2+^ signals to be lagging behind neuronal activation and that they were not required for ictogenesis (Baird‐Daniel et al. 2017). This may be a reflection of differences in epilepsy models, as (Baird‐Daniel et al. 2017) used 4‐Aminopyridine to elicit focal ictal activity. Reactive astrocytes have been shown to exhibit aberrant Ca^2+^ signaling in a number of epilepsy models, and astrocyte hyperactivity has been described as a key hallmark of epileptogenesis (Heuser and Enger 2021; Szokol et al. 2015). In a mouse seizure model, seizures caused rapid increases in Ca^2+^ levels of astrocytic endfeet, though this was confined to the seizure period (Tran et al. 2020). Several studies have reported continued increases in astrocytic Ca^2+^ signaling after the initial insult (Heuser et al. 2018; Shigetomi et al. 2019), although this could be a reflection of reactive gliosis, or the signals could be stimuli or stage specific. Ca^2+^ mediated astrocyte‐neuron interactions could be involved in the maintenance of neuronal hyperactivity, resulting in an excitatory feedback loop (Gómez‐Gonzalo et al. 2010; Henneberger 2017).

Aberrant astrocyte signaling in epilepsy could also be the result of altered gap junction communication (Henning et al. 2021). Connexins are tetraspan proteins that are the constituents of two channel types, gap junctions and hemichannels. Gap junctions are formed by two connexin hexamers that dock to each other on neighboring cell membranes (Walrave et al. 2020; Yang et al. 2021). Unpaired connexin hexamers can function as hemichannels, thus providing an additional means of astrocyte signaling (Xing et al. 2019). Astrocyte‐astrocyte communication is facilitated via gap junctions through direct cytoplasmic sharing. Gap junctions regulate synaptic activity and cerebrovascular blood flow, as well as facilitating the distribution of nutrients through the astrocytic syncytium and supply neurons with nutrients in an activity‐dependent manner (Mylvaganam et al. 2014). This means they are crucial for nutrient supply during the high energy demand during seizure activity (Rouach et al. 2008).

Connexin (Cx) 43 is highly expressed by astrocytes, and the most abundant connexin in the brain and hippocampus (Dermietzel et al. 1991; Rash et al. 2001). Cx43s have a complex and ambiguous role in epilepsy, where they are thought capable of suppressing seizure activity by redistributing K^+^ from areas of high activity (Kofuji and Newman 2004). Brief periods of epileptiform activity have been shown to result in uncoupling of gap junctions, resulting in reduced K^+^ clearance (Onodera et al. 2021). It has also been suggested that Cx43 gap junctions promote seizure activity by enhancing propagation of Ca^2+^ waves (Gómez‐Gonzalo et al. 2010), which could then lead to release of gliotransmitters from astrocytes thereby enhancing excitability. It is noteworthy that astrogliosis can also affect Cx43 expression and function (Mylvaganam et al. 2014) and that the inflammatory response following seizure activity exacerbated seizures through increased connexin hemichannel activity (Das et al. 2012). Pathogenic variants in the human Cx43 gene are also associated with seizures, as 30% of individuals with oculodentodigital dysplasia (ODDD)—a rare genetic disorder caused by mutations in the GJA1 gene, which encodes the Cx43 protein—experience neurological symptoms, including seizures (Barzegar et al. 2012). In tissue from AE patients, several studies reported upregulation of astrocytic Cx43 (Collignon et al. 2006; Das et al. 2012; Fonseca et al. 2002) but not neuronal connexins (Mylvaganam et al. 2010), whilst others observed no changes in AE models (Söhl and Willecke 2004; Walrave et al. 2020). Motaghi et al. (2017) found increased Cx43 protein in pilocarpine treated rats with focal seizures but not generalized seizures, whilst in a KA induced seizure model, astrocytic Cx43 was upregulated and neuronal Cx36 was downregulated, suggesting that astrocyte and neuronal connexin may regulate epileptiform activity differently (Vincze et al. 2019). Functional modifications such as altered phosphorylation may be more relevant than purely assessing mRNA or protein levels (Deshpande et al. 2017; Muñoz‐Ballester et al. 2024), where for example Cx43 phosphorylation has been described to contribute to seizure susceptibility following mild traumatic brain injury. Inhibition of connexin hemichannels has emerged as a potential therapeutic strategy as several drugs including the general connexin channel blocker carbenoxolone as well as more selective peptide inhibitors of hemichannels have been shown to attenuate seizure activity (Guo et al. 2022; Kiani 2023; Ran et al. 2018; Walrave et al. 2020).

## 3 Role of Astrocytic Glutamate Transporters in Acquired Epilepsy

Altered glutamate uptake and signaling is a common phenotype described in reactive astrocytes in neurological diseases with increased glutamate levels reported in patients with TLE and pre‐clinical TLE seizure models (Cavus et al. 2005; Peterson and Binder 2019). Astrocytes regulate extracellular glutamate via Na^+^ dependent transporters, glutamate aspartate transporter (GLAST) and glutamate transporter‐1 (GLT‐1), with human homologues being EAAT1 and EAAT2, respectively.

Although depletion of GLAST does not result in spontaneous seizures, experimentally triggered seizures were longer and more severe in knockout mice (Watanabe et al. 1999). Several acquired epilepsy models reported no changes in GLAST levels (Samuelsson et al. 2000; Tessler et al. 1999), although increased GLAST levels were reported by (Peterson and Binder 2020). GLT‐1 is predominantly expressed by astrocytes around synapses and conditional knock out of GLT‐1 resulted in reduced glutamate uptake ability of astrocytes (Petr et al. 2015). Deletion of GLT‐1 also led to spontaneous seizure activity (Sugimoto et al. 2018) and expression has been shown to be decreased in several models of epilepsy (Clarkson et al. 2020; Clasadonte et al. 2016; Hubbard et al. 2017; Samuelsson et al. 2000; Sarac et al. 2009; Ulu et al. 2010). Although changes in expression levels of these transporters have been reported in a number of epilepsy models, results have been variable. This could reflect different brain regions samples, time of data collection after seizure activity, whether neuron loss or gliosis is present and the severity thereof, or ischemia induced tissue proteolysis depending on the model.

Astrocytes also express G‐protein coupled metabotropic glutamate receptors (mGLURs), mGluR3 and mGluR5, that play a role in synaptic modulation (Aronica et al. 2000). In the developing brain, mGluR5 plays a role in glutamate transport, astrocyte motility and synapse ensheathment (Umpierre et al. 2019). Activation of mGluR5 has been shown to alter GLT‐1 activity and increase glutamate clearance (Higashimori et al. 2013; Morel et al. 2014), however, chronic activation has conversely been shown to result in reduced GLT‐1 and GLAST levels, and in turn reduced glutamate uptake (Aronica et al. 2003). In TLE patient and mouse models, upregulation of mGluR5 has been reported in astrocytes (Umpierre et al. 2019). After SE or application of a mGluR5 agonist, astrocytes re‐acquired mGluR5 dependent Ca^2+^ transients that are normally restricted to early development. Studies have suggested that activation of mGluR5 can lead to increased excitability by enhancing astrocytic Ca^2+^ signals. Activation of mGluR3 receptors is also thought to upregulate expression of glutamate transporters GLT‐1 and GLAST, thus increasing glutamate clearance and protecting neurons from excitotoxicity (Aronica et al. 2003; Zhou et al. 2006). In TLE models, both up and downregulation of mGluR3 has been reported so its role remains unclear (Peterson and Binder 2020).

Astrocytes not only play a role in modulating excitatory neurotransmission, but also inhibition. Reactive astrocytes may overproduce GABA (γ‐aminobutyric acid) in several forms of epilepsy (Müller et al. 2020; Pavlov and Walker 2013), which has been suggested to compensate for the loss of inhibitory neurons. GABA is thought to mediate neuronal excitability through either phasic inhibition, mediated by presynaptic GABA release, or tonic inhibition, through extracellular GABA that activates GABA receptors within the extra synaptic membrane of neurons (Bryson et al. 2020). Astrocytic GABA is thought to be produced through monoamine oxidase‐B and then released via the Best1 channel, shown to moderate tonic inhibition in several brain regions (Twible et al. 2021). Tonic inhibition by astrocytes affects brain function under physiological and pathological conditions; in a murine epilepsy model seizure activity was suppressed through Best1‐mediated tonic inhibition which inhibited Ca1 neuronal firing (Pandit et al. 2020). In a Best1^−/−^ mouse model, astrocyte specific expression of Best1 restored tonic inhibition but also seizure susceptibility (Pandit et al. 2020). Despite interneuron loss in a rodent model of TLE, no changes in tonic inhibition were apparent, thought to be due to astrocytic tonic signaling (Müller et al. 2020). Reactive astrocytes appeared to accumulate GABA, due to synthesis rather than uptake, which may reflect a compensatory mechanism to counteract hyperexcitability.

Both Glutamate and GABA synthesis relies on glutamine, and glutamine synthetase (GS) is the only enzyme capable of synthesizing large amounts of glutamine, serving crucial roles in nitrogen metabolism, acid–base homeostasis, and cell signaling. GS within the brain is highly expressed by astrocytes (Eid et al. 2020). In a number of AE mouse and rat models, region‐specific reduction of GS in astrocytes has been described (Bidmon et al. 2004; Van Der Hel et al. 2014; Lee et al. 2021; Setkowicz et al. 2017). Focal inhibition of GS in the hippocampus triggered epileptogenesis with gradual worsening of seizure severity (Albright et al. 2017). Inhibition of GS in astrocytes resulted in increased intracellular glutamate concentrations, release of which into the extracellular space could contribute to seizures and neuron loss (Perez et al. 2012).

It should be noted that astrocytes not only contribute to epilepsy but have also shown to be protective in in vitro models. For example, response times to pro‐convulsive agents were increased relative to the number of astrocytes in an in vitro model (Ahtiainen et al. 2021). More recently, it has been shown that optogenetic activation of astrocytes may attenuate seizures in a neocortical rodent model (Zhao et al. 2022).

Therefore it is clear that astrocytes play an important role in initiation and progression of acquired epilepsy (Figure 1), and hold significant potential as therapeutic targets (Çarçak et al. 2023; Dheer et al. 2022; Hayatdavoudi et al. 2022; Vezzani et al. 2022). However, the contribution of astrocytes to the pathogenesis of genetic epilepsies has not been well delineated. Here, we review the literature to date and discuss the potential of astrocytes as therapeutic targets in these disorders.

## 4 Genetic Epilepsies

Genetic epilepsies caused by single nucleotide pathogenic variants or copy number variants, including microdeletions/duplications, are individually rare but collectively common (Oliver et al. 2023). Advances in gene discovery have advanced our understanding of the complex mechanisms underlying the neurobiology of epilepsy (Ellis et al. 2020). The study of developmental and epileptic encephalopathies (DEEs) in particular has driven the discovery of de novo genetic pathogenic variants. Affected individuals commonly present with neurodevelopmental delay or regression. However, compared to AE, much less is known about how non‐neuronal cell types such as astrocytes contribute to neurological phenotypes, neuron dysfunction and seizure activity. Astrocytes play crucial roles in neurodevelopment, including regulating synaptic circuit development that affects formation, maturation and remodeling of synapses (Séjourné and Eroglu 2024). Perisynaptic astrocyte processes ensheathe synapses to modulate synapse formation and circuit plasticity, where a single astrocyte may contact thousands of synapses via extensively branched processes (Baldwin et al. 2024). Astrocyte‐dependent regulation of synapse formation and elimination is pivotal for refining synaptic connectivity, and alterations in these processes have been linked to aberrant neurodevelopment. During the period of postnatal development where synapses mature, astrocytes themselves undergo intense growth and structural changes. The role of astrocytes in neurodevelopment has been extensively covered in other reviews (Molofsk et al. 2012; Séjourné and Eroglu 2024; Vivi and Di Benedetto 2024), and here we will focus specifically on how pathogenic variants associated with genetic epilepsies impact astrocyte function.

Astrocytes play crucial roles in K^+^ homeostasis, where they transfer K^+^ from regions with high K^+^ levels, a process driven by spatial gradients in membrane potential and K^+^ equilibrium potential (Seifert et al. 2018; Steinhäuser et al. 2012). Epileptiform discharges have been shown to weaken syncytial coupling in astrocytes, impairing spatial K^+^ distribution (Wang et al. 2020). Pathogenic variants in several genes encoding potassium channels are prominent causes of DEE, including KCNQ2, which encodes the K_V_7.2 subunit of K_V_7 potassium channels. In a human RNA sequencing data set, KCNQ2 was found to be highly expressed in foetal astrocytes (17–20 gestational weeks) or astrocyte precursor cells but not mature astrocytes (Bottom‐Tanzer and Dulla 2022; Zhang et al. 2016), suggesting that it may be restricted to the early neurodevelopmental period (Dirkx et al. 2020). However, in a conditional transgenic mouse model carrying a KCNQ2 patient variant that exhibits spontaneous seizures (Bottom‐Tanzer and Dulla 2022), an increased number of astrocytes were observed as well as widespread upregulation of GFAP (Kim et al. 2021), consistent with a reactive astrocyte phenotype. In these adult mice, K_V_7.2 was also detected in GFAP positive cells, although to a lesser extent than in neurons.

Inward rectifying K^+^ channels (Kir) are highly expressed by astrocytes, where in mouse and human hippocampal astrocytes the main subunit is Kir4.1 (, Olsen and Sontheimer 2008, Seifert et al. 2009). Kir4.1 channels have weakly rectifying properties that allow bidirectional K^+^ movement, aiding in spatial K^+^ buffering (Olsen and Sontheimer 2008) and are responsible for the negative resting membrane potential (−85 mV) of astrocytes (Dibaj et al. 2007). EAST syndrome, also known as SeSAME syndrome, is an autosomal recessive disorder caused by pathogenic variants in the *KCNJ10* gene, which encodes the potassium channel Kir4.1 (Abdelhadi et al. 2016). First described in 2009, clinical symptoms of EAST syndrome include epilepsy, ataxia, sensorineural deafness, developmental delay and electrolyte imbalance (Bockenhauer et al. 2009, Scholl et al. 2009). These pathogenic variants have been associated with a loss of function of Kir4.1 that is replicated in a zebrafish loss of function model (Mahmood et al. 2013), however, potential gain of function mechanisms have been described (Sicca et al. 2016). Given the role of Kir4.1 in astrocytes, it is likely that pathogenic variants in KCNJ10 lead to altered potassium buffering in astrocytes. In astrocytoma cell lines that express either WT or mutant Kir4.1 channels, whole cell current densities were significantly larger in mutant cells enhancing K^+^ influx (Sicca et al. 2016). Whilst WT Kir4.1 was expressed around the soma, the mutant channel was located along cell membranes. Overall, expression of mutant Kir4.1 was more abundant than WT Kir4.1, alongside upregulation of a‐syntrophin. The appropriate distribution and density of Kir4.1 in discrete astrocytic subdomains is crucial for astrocyte mediated K^+^ buffering to maintain optimal K^+^ concentrations in the extracellular environment, whilst alterations may impact synapse functionality. In an astrocyte‐conditional Kir4.1^−/−^ mouse model, astrocytes exhibited changes in their whole‐cell current pattern and were more depolarised than WT astrocytes (Djukic et al. 2007). Blocking Kir channels with Barium (Ba^2+^) resulted in similar depolarisation in WT astrocytes. Furthermore, both K^+^ and glutamate uptake was impaired in Kir4.1^−/−^ astrocytes. In the same mouse model, spontaneous neuronal activity was reduced in CA1 pyramidal neurons in acute hippocampal slices. Whilst baseline synaptic transmission was unaltered in hippocampal neurons following stimulation (0–80 μA), investigating long term potentiation revealed a greater potentiation for 20 min after the initial pulses. This suggests that Kir4.1 may play a role in clearing K^+^ levels following increased synaptic activity rather than under basal synaptic transmission (Djukic et al. 2007). Altered ion‐water homeostasis has also been described in EAST syndrome patients where intramyelinic edema were observed, likely driven by abnormal function of Kir4.1 channels at astrocyte endfeet (Severino et al. 2018).

Furthermore, single nucleotide polymorphisms (SNPs) in Kir4.1 have been reported in TLE patients (Heuser et al. 2010), whilst pathogenic variants in Kir4.1 in mice increase seizure susceptibility (Buono et al. 2004; Ferraro et al. 2004; Inyushin et al. 2010; Nwaobi et al. 2016; Tong et al. 2014). Changes in Kir channel function and reduced expression have been reported extensively in AE (Harada et al. 2013; Heuser et al. 2010; Kinboshi et al. 2019; Steinhäuser et al. 2012), and several antiepileptic drugs have been shown to increase Kir4.1 expression in astrocytes (Kinboshi et al. 2019; Mukai et al. 2018). Further research is needed to establish what drives seizure activity in EAST syndrome, or how astrocytes carrying Kir4.1 pathogenic variants affect neuronal function.

Pathogenic variants in solute carrier family 6 member 1 (*SLC6A1*), encoding GABA transporter 1 (GAT1) are associated with a range of neurodevelopmental disorders, including childhood absence epilepsy, epilepsy with myoclonic atonic seizures and Lennox Gastaut syndrome. GAT‐1 is expressed by both inhibitory neurons and astrocytes (Minelli et al. 1995), and a direct correlation between astrocytic GAT‐1 deficit and absence seizures has been reported (Crunelli et al. 2020; Pirttimaki et al. 2013). GABA uptake was significantly reduced in primary mouse astrocytes transfected with a patient variant (Mermer et al. 2022; Poliquin et al. 2021), primary astrocytes isolated from SLC6A1 knock in mice (Nwosu et al. 2022) and patient induced pluripotent stem cell (iPSC) derived astrocytes (Mermer et al. 2021, 2022; Nwosu et al. 2022). GABA uptake could be restored by treatment with 4‐phenylbutyrate (Nwosu et al. 2022). In primary murine and iPSC derived astrocytes carrying GAT‐1 variants associated with epilepsy, reduced expression and altered distribution of GAT‐1 was observed with increased expression around the nucleus and little expression at the periphery of the cells (Mermer et al. 2021, 2022), suggesting retention in the ER. Mutant GAT‐1 appeared aggregated in astrocytes, with changes in GAT‐1 glycosylation that could result in different amounts of mature protein at the cell surface (Mermer et al. 2021). WT GAT‐1 expressed in variant carrying iPSCs co‐localized with the ER, suggesting a dominant effect (Nwosu et al. 2022). Tonic inhibition by astrocytes affects brain function under physiological and pathological conditions (Héja et al. 2012), and reduced GABA uptake by GAT‐1 deficient astrocytes could result in extracellular GABA accumulation and enhanced tonic inhibition. Enhanced tonic inhibition in the thalamus has been shown to result in absence epilepsy and spike wave discharges in multiple animal models (Mermer et al. 2022; Pirttimaki et al. 2013). Astrocyte specific expression of *SLC6A1* variants could provide further evidence of how changes in tonic inhibition drive seizures in SLC6A1 patients, or the relative contribution of astrocytes to seizure and neurodevelopmental phenotypes.

SLC13A5 is an inward‐directed electrogenic sodium coupled tricarboxylate substrate transporter and pathogenic variants in *SLC13A5* can result in a citrate transporter disorder leading to DEE with amelogenesis imperfecta (Thevenon et al. 2014). It is highly expressed in adult astrocytes (Zhang et al. 2014, 2016) and shows high affinity to citrate, albeit in lower levels compared to neurons (Kumar et al. 2021). Elevation of citrate and dysregulation of citric acid cycle intermediates in plasma, CSF and urine of patients (Bainbridge et al. 2017), suggest dysregulation of the mitochondrial tricarboxylic acid (TCA) cycle. This is highly dependent on astrocytes as citrate is released by astrocytes and taken up into neurons via SLC13A5 (Sonnewald et al. 1991). Henke et al. (2020) found increased citrate and decreased lactate levels in the CSF of SLC13A5^−/−^ mice, indicating that elevated citrate may inhibit glycolysis in astrocytes.

Dravet syndrome (DS) is a severe DEE, where over 90% of cases are caused by a haploinsufficiency of the *SCN1A* gene. *SCN1A* encodes Na_V_1.1, the alpha subunit of a voltage gated sodium channel (Brunklaus et al. 2014). Whilst *SCN1A* is highly expressed in neurons, it is also expressed in glial cells including astrocytes. Reactive astrocytes and microglia were observed in retinal tissue from a DS knock in mouse (Salazar et al. 2023). In a mouse model of DS that carries a heterozygous mutation and replicates disease phenotypes, including seizure activity, astrocyte GFAP intensity and the number of GFAP+/S100b+ astrocytes were significantly increased (Martín‐suárez et al. 2020). Astrocyte morphology appeared to be more complex in DS astrocytes, consistent with increased astrocyte reactivity (Martín‐suárez et al. 2020; Valassina et al. 2022). Interestingly, Goisis et al. (2022) observed increased GFAP expression in entorhinal cortex and dentate gyrus of DS mice prior to seizure onset. Increased astrocyte proliferation was reported in mice carrying a missense pathogenic variant in *SCN1A* (Satta et al. 2021) and in a zebrafish model (Tiraboschi et al. 2020), where scRNA sequencing revealed enrichment for gliosis related genes. Valassina et al. (2022) observed increased expression of pan‐reactive markers Vimentin and GFAP in their mouse RNA sequencing data set in both cortical and hippocampal samples. Investigation of A1 “neurotoxic” and A2 “neuroprotective” markers in astrocytes, revealed that markers from both sets were upregulated in DS mice, but A2 markers more so (Valassina et al. 2022), highlighting that astrocytes cannot be neatly divided into A1 and A2 categories. Expression of these genes were restored to control levels when SCN1A expression was restored in Cre mice. These studies highlight that astrocytes adapt a reactive phenotype in DS, however it is unclear how this affects seizure activity and neuronal function. Uchino et al. (2023) reported that in a mouse with a loss of function pathogenic variant in *SCN1A*, Ca^2+^ signaling was altered, with an increased slope of spontaneous and ATP‐induced Ca^2+^ spiking, as well as increased ATP‐induced transient Ca^2+^ influx. It is possible that similar to AE, Ca^2+^ mediated astrocyte‐neuron interactions could be involved in the maintenance of neuronal hyperactivity, resulting in an excitatory feedback loop. Further research is needed in DS, to establish which astrocytic phenotypes are cell autonomous and how astrocyte‐neuron interactions are impacted.

Lennox Gastaut syndrome is a childhood onset DEE characterized by multiple seizure types (Asadi‐Pooya 2018) that has been linked to numerous genetic aetiologies including *ALG13*, which is highly expressed in neurons (Gao et al. 2019). In serum from patients with Lennox Gastaut syndrome, elevated levels of YKL‐40, which can be secreted by reactive astrocytes among other cell types, were reported, although other markers such as GFAP were not increased (Cherkezzade et al. 2024). There is conflicting evidence as to whether ALG13 is expressed in astrocytes (Alsharhan et al. 2021; Huo et al. 2020). Although *ALG13* ^*−/−*^ mice showed increased astrocytic responses to KA induced seizures compared to wild‐type mice (Gao et al. 2019), including increased GFAP positive astrocyte number, hypertrophy and glial scar formation around areas of neuronal loss, this may be a reflection of greater neuronal seizure activity and loss in *ALG13* ^*−/−*^mice.

*WWOX*‐related DEE is caused by variants in tumor suppressor WW domain‐containing oxidoreductase (*WWOX*). Wwox^−/−^ mice showed evidence of astrogliosis and upregulation of cytokines TNF‐a and IL‐6 (Hussain et al. 2019), although no seizure activity was reported. However, similar astrocyte reactivity and upregulation of pro‐inflammatory pathways were observed in a WWOX‐loss of function mouse model that exhibited spontaneous seizures (Hussain et al. 2023). Steinberg et al. (2021) generated 3D brain spheroids from induced pluripotent stem cells edited to carry a patient variant. They noted a marked increase in astrocytes stained for GFAP and S100b, which could be partially reversed by overexpression of WWOX. Similarly, gene expression of S100b, GFAP, AQP4, and ALDH1A1 was significantly upregulated by week 24 in mutant cerebral spheroids, which could be restored to wild type levels by WWOX overexpression. RNA sequencing revealed upregulation of a number of astrocyte genes including *AGT*, *S100A1*, *GJA1*, and *OTX2*. Whilst Repudi et al. (2021) found WWOX to be expressed in astrocytes, they did not observe developmental delay, weight loss, or postnatal lethality in mice where WWOX was conditionally knocked out in astrocytes. However, neuron conditional knockout WWOX mice did show evidence of astrocyte reactivity including upregulation of GFAP (Repudi et al. 2021), indicating that changes in astrocyte phenotypes occur as a result of neuronal dysfunction and seizure activity, similar to what has been described in AE (Vezzani et al. 2022).

*SCN8A*‐related DEE is caused by pathogenic variants in voltage‐gated sodium channel Na_V_1.6, which plays a pivotal role in controlling neuronal excitability. In a TALEN‐generated knock‐in mouse model expressing a patient *SCN8A* variant, upregulation of GFAP in hippocampal astrocytes was observed following seizure activity (Sprissler et al. 2018). RNA sequencing performed on mouse brains revealed that changes in gene expression were only observed after seizure activity (Sprissler et al. 2018). A significant number of differentially expressed genes were associated with reactive astrocytes and matched changes observed in brain injury, ischemia, and neuroinflammation. Similarly, following spontaneous seizures in an Scn8a mouse model carrying a patient missense mutation (D/+ mice) (Thompson et al. 2022), the number of GFAP+ astrocytes increased in both cortex and hippocampus, whilst total astrocyte number remained the same. However, expression of glutamine synthetase (GS) in astrocytes was decreased, which has also been reported in AE and correlated with neuronal loss (Van Der Hel et al. 2014; Lee et al. 2021; Setkowicz et al. 2017). Changes in K^+^ homeostasis were also observed in (D/+) mice (Thompson et al. 2022). In young mice, prior to seizure activity, a trend towards increased Kir4.1 current and amplitude was observed suggesting that altered astrocyte function may precede seizure activity. Conversely, in older mice that exhibited spontaneous seizures, astrocytes exhibited reduced Kir4.1 currents and a reduction in amplitude, indicative of disruption in astrocytic K^+^ buffering (Thompson et al. 2022). Although Na_V_1.5 is the more highly expressed voltage‐gated sodium channel in astrocytes, some studies have found evidence of Na_V_1.6 expression (Liu, Zhang, et al. 2023; Reese and Caldwell 1999; Zhu et al. 2016, 2017). Thompson et al. did not find overlap of Na_V_1.6 with astrocyte markers, albeit in D/+ mice where Na_V_1.6 expression may have been altered. In KA and PTZ‐induced seizure models, Na_V_1.6 was upregulated in a seizure intensity‐dependent manner in reactive astrocytes (Zhu et al. 2016), suggesting that astrocytes may facilitate hyperexcitability.

Glucose transporter 1 (Glut‐1) deficiency syndrome (G1D) is caused by *SLC2A1* haploinsufficiency and characterized by infantile seizures, movement disorder, and developmental delay (Kim et al. 2013). Pathogenic variants in *SLC2A1* can result in severe DEE but also early onset absence epilepsy. Astrocytes take up glucose from peripheral blood through GLUT‐1 localized at astrocytic endfeet where it is synthesized to glycogen (Brown and Ransom 2007). Astrocytic glucose plays a crucial role in lactate synthesis, which is supplied to neurons. G1D mice showed substantial alterations in brain metabolism, including depletion of glucose, decreased brain acetyl‐CoA, and fatty acids (Marin‐valencia et al. 2012). However, components of the TCA were unaffected, potentially balanced by elevated ketone body levels. In primary astrocyte cultures prepared from heterozygous SLC2A1 mice, valproic acid (VA) increased glucose uptake, potentially through increasing histone acetylation of the SLC2A1 promoter and thus increasing gene expression (Kim et al. 2013). This contradicts earlier work (Wong et al. 2005) which suggested that VA reduced glucose transport in healthy primary astrocytes. G1D mice also showed evidence of astrocytosis (Marin‐valencia et al. 2012; Ullner et al. 2009), with increasing reactivity as mice aged; however, it remains unclear how G1D astrocytes impact neuronal health.

Pyridoxine‐dependent epilepsy (PDE) is a rare autosomal recessive disease caused by pathogenic variants in the *ALDH7A1* gene, with some evidence suggesting astrocyte involvement. *ALDH7A1* encodes the enzyme antiquitin, which is highly expressed in glial cells. Gliosis of cortical astrocytes was reported in one post‐mortem brain of a patient with a pathogenic ALDH7A1 variant (Jansen et al. 2014; Johal et al. 2024). Although gliosis was absent in a knockout mouse model (Al‐Shekaili et al. 2020), in primary astrocytes from an *Aldh7a1* ^−/−^ mouse model (Johal et al. 2024), accumulation of saccharopine, P6C, and pipecolate was observed compared to control astrocytes, which suggests deficiencies in lysine metabolism. Astrocyte‐specific deletion of *ALDH7A1* in mice resulted in PDE phenotypes, including spontaneous seizure activity, abnormal dendritic spine development, and synaptic transmission, alongside cognitive impairments (Wu et al. 2025). This is thought to be caused by dysregulation of astrocyte‐derived matrix gla protein (MGP), a vitamin K‐dependent protein, as supplementation of vitamin K rescued neuronal phenotypes.

Biallelic variants in *SLC25A22*, which encodes a mitochondrial glutamate carrier (solute carrier family 25, mitochondrial carrier, glutamate, member 22) cause a severe infantile DEE. *SLC25A22* is more abundant in astrocytes than in neurons (Berkich et al. 2007), and facilitates glutamate transport into the mitochondrial matrix. In primary astrocyte mono‐cultures (Goubert et al. 2017), where SLC25A22 was silenced with shRNA, reduced nicotinamide adenine dinucleotide (Phosphate) (NAD(P)H) formation was observed after glutamate stimulation compared to control cultures. In shRNA treated astrocytes, inactivation of SLC25A22 resulted in cytosolic glutamate accumulation, demonstrating the importance of this carrier in astrocytic glutamate homeostasis. Further, high intracellular concentrations of glutamate are shown to reduce extracellular glutamate uptake (Barbour et al. 1988; Otis and Jahr 1998; Bergles and Jahr 1998; Grewer and Rauen 2005; Wadiche et al. 2006; Tzingounis and Wadiche 2007). Other causes of mitochondrial epilepsy, including POLG, show mitochondrial respiratory chain defects in astrocytes, including reduced expression of complexes I and IV (Chan et al. 2019). Reduced glutamine synthetase expression was observed in patients with mitochondrial disease and epilepsy, but not in those with mitochondrial disease without epilepsy. In Alper's syndrome, a childhood‐onset mitochondrial disease with refractory epilepsy caused by bi‐allelic pathogenic variants in POLG, hypertrophic, GFAP‐reactive astrocytes were present in the primary visual cortex. This subpopulation of astrocytes also exhibited decreased abundance of mitochondrial complex I subunit NDUFB8 and complex IV subunit COXI, reduced GS staining whilst expression of AQP4 and Kir4.1 was increased (Smith et al. 2023).

Several other genes associated with genetic DEE are expressed in astrocytes, albeit at lower levels compared to neurons and further research is needed to establish whether these affect astrocyte function and how this contributes to pathogenesis. For example, pathogenic heterozygous variants in *SPTAN1* have been associated with DEE including infantile epileptic spasms syndrome (IESS) (Tohyama et al. 2008). Chun et al. (2021) showed that human astrocytes secrete SPTAN1 in extracellular vesicles to modulate neuronal survival and electrophysiological function. Mice with a predominantly astrocyte specific TCF4 heterozygous knockout exhibited recurrent seizure activity and premature mortality (Craciun et al. 2025). Astrocyte reactivity was observed in several brain regions with increased neuronal activity and widespread transcriptional changes in astrocytes, neurons and oligodendrocytes. De novo variants in SIK1 have been found in a number of DEEs, including infantile epileptic spasms syndrome. SIK1 expression was not only present in neurons, but also cortical white matter astrocytes (Hansen et al. 2015). Whilst the role of these genes in astrocytes remains unclear, there is potential that variants may affect astrocyte function and impact astrocyte responses to neuronal seizure activity.

**FIGURE 2:**
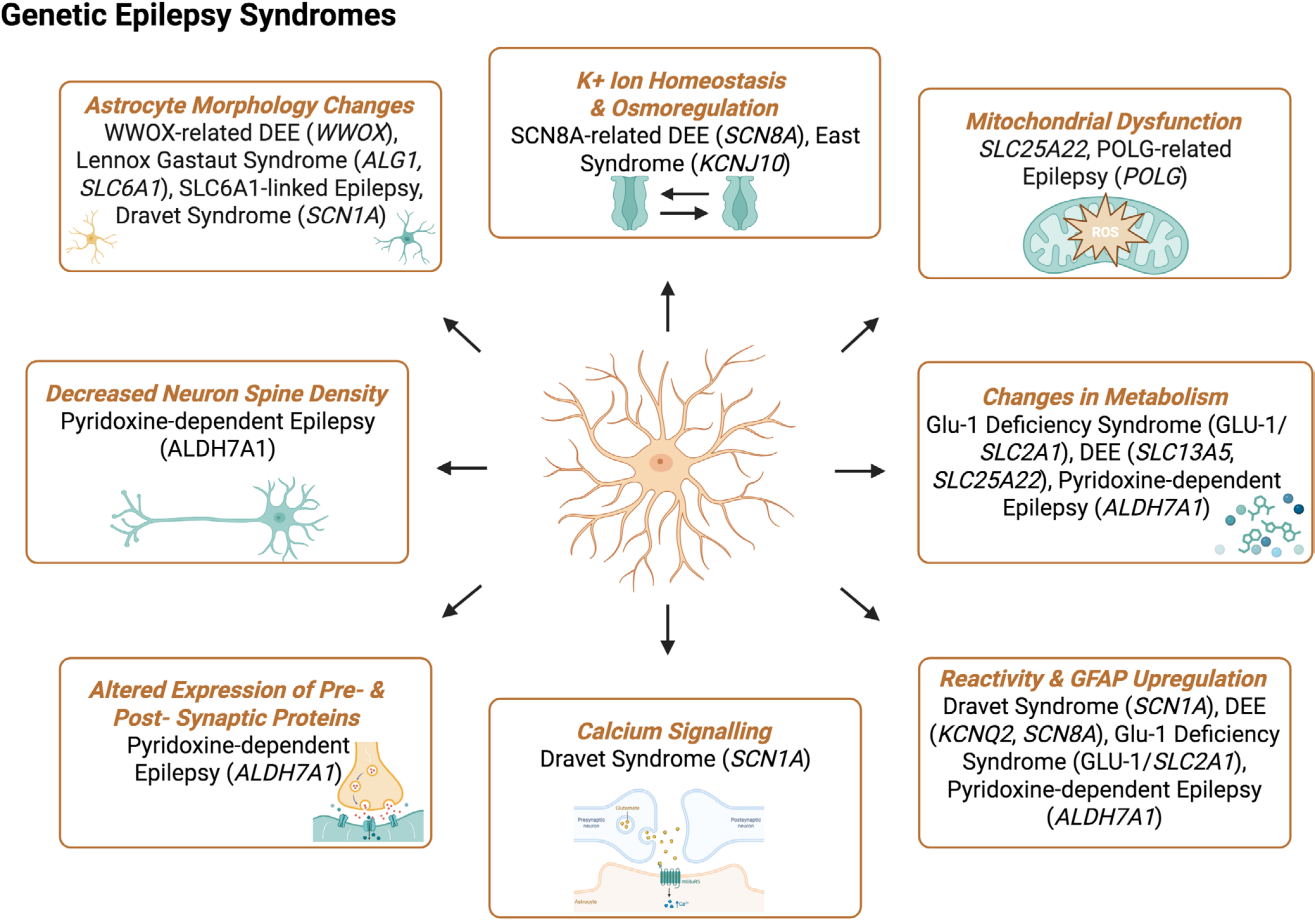
Altered astrocyte function in genetic epilepsies.

While substantial progress has been made in identifying pathogenic variants that cause genetic epilepsies, most studies to date have focused on neuronal phenotypes, in part due to the genes being highly expressed in neurons. However, the studies discussed above highlight the importance of understanding the contribution of astrocytes to disease pathophysiology (Figure 2). Given the potential of targeting astrocytes for novel treatments in AE, their role in worsening seizure activity and neuronal hyperexcitability, astrocytes remain an underexplored area in genetic epilepsies.

## 5 Neurodevelopmental Disorders

Neurodevelopmental disorders are a broad group of disorders with impaired cognition, social interactions, and language development. They are often accompanied by epilepsy, with up to 26% of patients experiencing seizures (Gonzalez‐Sulser 2020). Several neurodevelopmental disorders are characterized by aberrant astrocyte function in addition to neuronal dysfunction, with similar phenotypes as those described in epilepsy. Here, we will briefly discuss astrocyte dysfunction in childhood neurodevelopmental disorders that are associated with epilepsy.

Fragile X syndrome (FXS) is a monogenic neurodevelopmental disorder caused by a trinucleotide repeat expansion on the *FMR1* gene, which results in silencing of the gene and loss of protein function. Approximately 20% of FXS patients also have epileptic seizures. Astrocytes express *FMR1*; culturing *FMR1* ^−/−^ astrocytes with wild type neurons resulted in abnormal neuronal morphology in addition to decreased expression of pre‐and postsynaptic proteins (Jacobs and Doering 2010). Astrocytes derived from FXS patient iPSCs and *FMR1* ^−/−^ knock out human embryonic stem cells (hESCs), also exhibited altered cell cycle dynamics, consistent with increased astrocyte number in patient post mortem tissue (Ren et al. 2023). Enhanced Ca^2+^ signaling in response to ATP application was also observed in iPSC‐derived astrocytes, indicative of astrocyte hyperexcitability in FXS. Proteomic analysis revealed significant changes in protein expression, notably in metabolic pathways, whilst cholesterol metabolism was functionally impaired (Ren et al. 2023). Defects in purinergic signaling were apparent in primary *FMR1* ^−/−^ astrocytes, including differences in intracellular stores of UDP, ATP, AMP and adenosine, as well as significantly increased extracellular levels of adenosine in *FMR1* ^−/−^ astrocyte media (Reynolds, Napier, et al. 2024). Upregulation of purinergic receptors in *FMR1* ^−/−^ astrocytes had previously been reported, whilst application of ATP and UTP evoked greater Ca^2+^ responses than in WT astrocytes, suggesting a role for purinergic signaling in aberrant *FMR1* ^−/−^ astrocyte activation (Reynolds et al. 2021). Intriguingly, evidence suggests that FMRP mediates synaptic connectivity via astrocytes. Astrocyte conditioned media from primary *FMR1* ^−/−^ cultures enhanced neurite extension and complexity in WT and *FMR1* ^−/−^ neurons, whilst in *FMR1* ^−/−^ astrocyte‐neuron co‐cultures neuronal firing was enhanced, but associated with deficits in firing synchronicity (Reynolds, Huang, et al. 2024). These defects could be normalized by application of a selective P2Y_2_ purinergic receptor antagonist. In an astrocyte conditional *FMR1* ^−/−^ knockout mouse model, loss of FMRP in astrocytes led to cortical hyperactivity, as well as behavioral abnormalities in mice (Jin et al. 2021) which could be rescued by re‐expression of *FMR1* in astrocytes. FXS astrocytes, differentiated from patient derived iPSCs, induced increased neuronal excitability in both patient and control cortical neurons with a higher number of spontaneous bursts of action potentials lasting a shorter duration, whilst FXS neurons co‐cultured with control astrocytes did not exhibit aberrant burst firing (Sharma et al. 2023). The aberrant firing pattern was associated with reduced persistent sodium currents, and enhancing these currents normalized burst activity. Moreover FXS astrocyte conditioned media was sufficient to induce the burst firing phenotype, whilst addition of astrocyte protein S100b to cultures restored normal firing activity. Bataveljic et al. (2024) found an activity‐dependent increase in extracellular K^+^ levels in the hippocampus of a *FMR1* ^−/−^ knockout mouse model and established that *FMR1* ^−/−^ astrocytes exhibited reduced peak amplitude of Kir4.1 currents. Furthermore, expression of Kir4.1 expression was lower in hippocampal *FMR1* ^−/−^ astrocytes in the absence of morphological changes. In WT mice, Kir4.1 mRNA co‐localized with FMRP, suggesting that Kir4.1 is regulated by FMRP. Viral delivery of Kir4.1 not only rescued activity dependent K^+^ uptake in *FMR1* ^−/−^ astrocytes but also restored neuronal excitability and rescued behavioral deficits in *FMR1* ^−/−^ knockout mice. This not only highlights the role of astrocytes in increasing neuronal excitability in FXS but also emphasizes astrocyte function as a crucial therapeutic target.

Rett syndrome, a severe childhood neurodevelopmental disorder associated with developmental regression, motor stereotypies, autistic features and seizures, is caused by pathogenic variants in transcriptional repressor gene *MECP2*. MeCP2 is expressed in astrocytes (Ballas et al. 2009; Kifayathullah et al. 2010; Nagai et al. 2005; Pejhan et al. 2020; Zachariah et al. 2012), with lower expression in astrocytes from post mortem samples from Rett patients compared to unaffected individuals (Pejhan et al. 2020). Lowering of MeCP2 expression was also associated with aging in a murine MeCP2^+/−^model. The disease presents with a number of astrocytic phenotypes (Kahanovitch et al. 2019), however in contrast to other disorders with epilepsy, reactive gliosis is not a common phenotype (Kahanovitch et al. 2018; Pacheco et al. 2017). Dysregulated cholesterol metabolism has been linked to Rett syndrome, and elevated cholesterol levels have been found in serum samples from Rett syndrome patients (Segatto et al. 2014, 2019). Whilst these have not directly been linked to astrocytes, astrocytes synthesize the majority of brain cholesterol (Björkhem and Meaney 2004). hPSC‐derived astrocytes with *MECP2* pathogenic variants exhibited widespread transcriptional changes, as well as impaired metabolic homeostatic activity, possibly driven by mitochondria dysfunction (Sun et al. 2023). Rett astrocytes also displayed changes in mitochondrial metabolism, morphology and increased ROS generation (Bebensee et al. 2017; Tomasello et al. 2024). Mitochondria isolated from MECP2 mutant hESC‐derived astrocytes increased local field potential activity in cortical neurons, suggesting that astrocyte mitochondrial dysfunction in Rett syndrome may directly affect neuronal activity (Tomasello et al. 2024). MeCP2 modulates a number of target gene transcripts in astrocytes (Yasui et al. 2013), including GFAP in the developing brain (Forbes‐lorman et al. 2014). Kir4.1 is a direct target of MeCP2, and astrocytes from Mecp2^−/−^ mice express less Kir4.1 mRNA and protein, as well as changes in Kir4.1 mediated currents (Kahanovitch et al. 2018), which was also observed in isolated astrocytes indicating a cell autonomous phenotype. MeCP2 deficiency also appears to affect cell signaling to a substantial extent. Astrocytes obtained from MeCP2^−/−^ mice exhibited abnormal glutamate clearance and changes in EAAT1/EAAT2 expression, as well as increased levels of glutathione protein (Okabe et al. 2012). Interestingly, in WT astrocytes co‐cultured with mosaic MeCP2^+/−^astrocytes MeCP2 expression significantly decreased in a time dependent manner (Maezawa et al. 2009). This appeared to be facilitated via gap junctions, as inhibition of gap junctions by gap junction inhibitors carbenoxolone and 18α‐glycyrrhetinic acid, as well as Cx‐43 siRNA reversed the reduced MeCP2 expression pattern in WT astrocytes.

Changes in astrocyte morphology have also been reported (Sun et al. 2023) which could partially be rescued by the presence of WT neurons or astrocytes. Indeed, neuron–glia interactions appear to drive a number of phenotypes in *MECP2* Rett Syndrome. Abnormal Ca^2+^ activity in astrocytes led to excessive activation of extra synaptic NMDA receptors, increasing network excitability in Mecp2^−/−^ mice, indicating that abnormal Ca^2+^ signaling is a key cell‐autonomous phenotype (Dong et al. 2018). Interestingly, deletion of MeCP2 specifically either in astrocytes or all cells in Rett mice led to significantly reduced tonic inhibition in pyramidal neurons. Increases in GAT3 were apparent alongside increased GAT3 current in hippocampal astrocytes (Dong et al. 2020). MECP2 mutant mice had a lack of synaptic response to astrocytic stimulation compared to WT mice (Rakela et al. 2018). Primary WT neurons co‐cultured with Mecp2^−/−^ astrocytes displayed reduced synaptic puncta, which interestingly affected cortical but not hippocampal or cerebellar astrocytes. In these co‐cultures, decreased postsynaptic currents were also observed (Albizzati et al. 2024). Transcriptomic analysis of Mecp2^−/−^ astrocytes revealed differential expression of genes associated with synapse maturation, whilst inflammatory pathways were upregulated in WT neurons co‐cultured with Mecp2^−/−^ astrocytes, which appeared to be driven by increased expression of IL‐6 by astrocytes. Notably, increased astrocytic IL‐6 expression was only apparent in co‐cultures. Inhibition of IL‐6 rescued pre‐synaptic puncta formation, but only partially restored post‐synaptic puncta. Taken together, these data suggest astrocytes make a significant contribution to neuronal dysfunction and hyperexcitability in *MECP2* Rett Syndrome.

Duplication 15q syndrome (Dup15q) is caused by duplications of the maternal copy of chromosome 15q11‐q13 leading to pharmacoresistant epilepsy and autistic spectrum disorder. Additional copies of E3 ubiquitin ligase (*UBE3A*) lead to neuronal phenotypes, resulting in hyperexcitable neurons (Fink et al. 2021). Expression of UBE3A in astrocytes has been reported, although at lower levels than neurons (Gonzalez Ramirez et al. 2024). Differences in UBE3A expression in astrocyte subpopulations were observed, with a small subpopulation of astrocytes expressing lower UBE3A levels, suggesting that specific subpopulations of astrocytes may be affected. Notably, in drosophila, overexpression of the fly Ube3a homolog in glia, but not in neurons, caused seizure‐like phenotypes, altered neuronal morphology, and reduced K^+^ levels in glial cells (Hope et al. 2017). Transcriptomic and proteomic profiling in this glial specific Dup15q model revealed a cell autonomous upregulation of glutathione S‐transferase (GST) genes in glial cells, as well as enrichment of synaptic genes (Hope et al. 2020). These flies were susceptible to mechanical stress (“bang”) induced seizures. The authors identified other seizure susceptible drosophila lines with “gliopathic” epilepsy, all of which showed upregulation of GST genes. This work highlights a role for glial cells in epileptogenesis in Dup15q.

## 6 Conclusion

**FIGURE 3:**
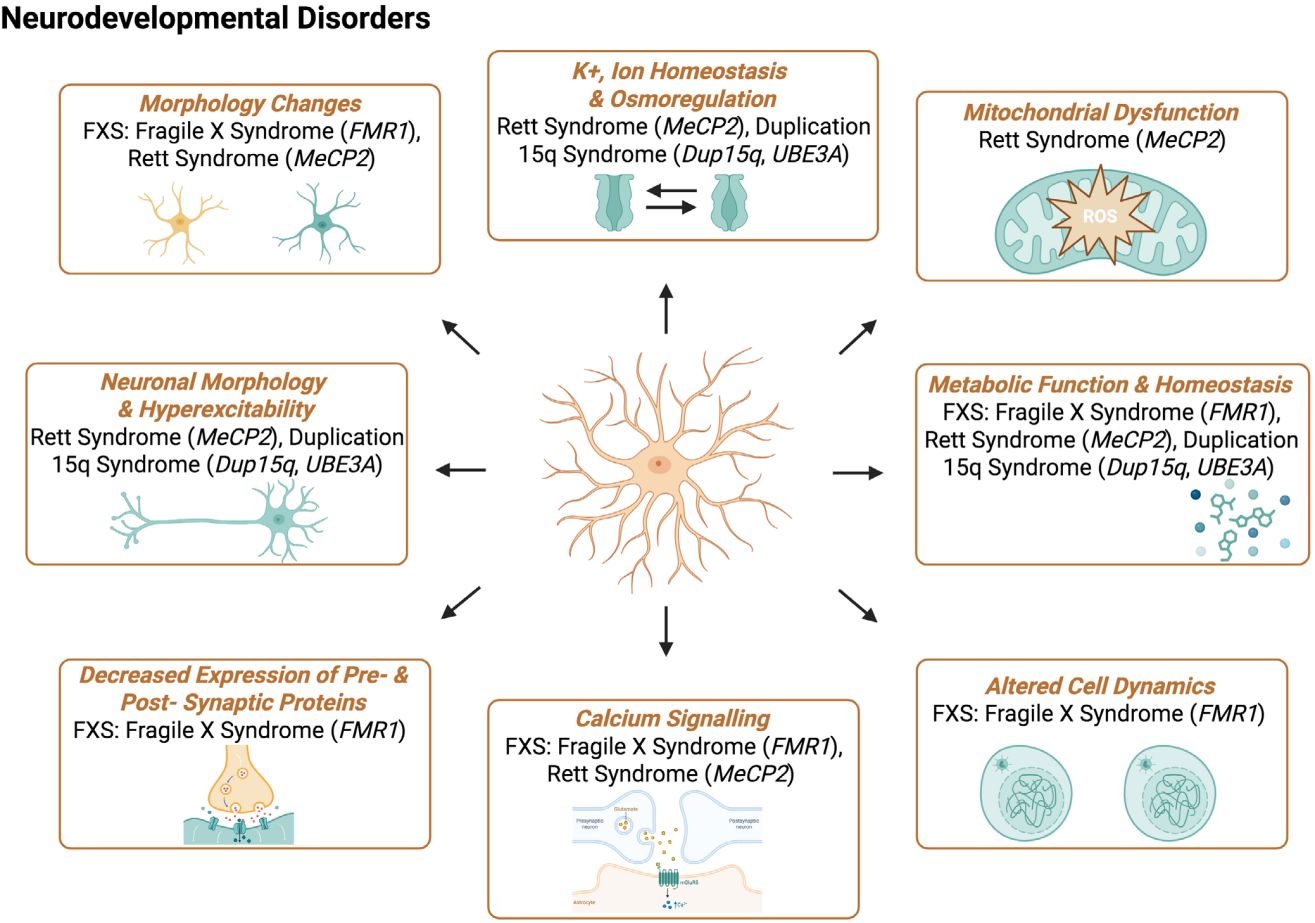
Astrocyte dysfunction in neurodevelopmental disorders with an epileptic component.

Emerging evidence suggests that astrocytes play a significant role in neurodevelopmental and neurological disorders of childhood (Figures 2 and 3). It is clear that astrocytes fulfill many key homeostatic functions, and that these are commonly disrupted in neurological disorders such as epilepsy. Common astrocyte phenotypes include modulation of inflammatory pathways, regulation of metabolites, glutamate clearance, and altered Ca^2+^ signaling. Astrocytes display pronounced responses to seizure activity, most notably astrocyte reactivity, but may also contribute to the initiation of seizure activity. This may occur through cell autonomous actions, or interactions of astrocytes, neurons, and other glial cells such as microglia. Due to the complex and interdependent networks between glial cells and neurons, it can be difficult to establish the relative contribution of astrocytes. Neuron‐astrocyte co‐culture models, using murine or iPSC‐derived cells, can elucidate the contributions of astrocytes to neuronal seizure activity and have the potential to uncover underlying mechanisms (Liu et al. 2022; Peng et al. 2021; Peters 2021). The emergence of more complex iPSC‐derived organoid models that include microglia or vasculature, as well as assembloids that integrate organoids modeling several brain regions, may also serve as more physiological models to study astrocyte‐neuron interactions in genetic epilepsies (Bagley et al. 2017; Lange et al. 2022; Marton et al. 2019; Nzou et al. 2018; Sloan et al. 2017).

The contribution of astrocytes to the pathophysiology and clinical manifestations of genetic epilepsies and neurodevelopmental disorders remains incompletely understood, partly due to the predominantly neuron‐centric focus of research to date. As we have detailed in this review, accumulating evidence indicates astrocyte dysfunction represents an important and underappreciated component of disease mechanisms, and astrocyte pathways represent a viable target for the development of innovative epilepsy treatments (Çarçak et al. 2023; Chen et al. 2024; Shen et al. 2023). A more comprehensive understanding of astrocyte involvement in genetic epilepsies would provide new insights into disease pathogenesis and identify additional therapeutic opportunities.

## Declaration of Transparency

The authors, reviewers and editors affirm that in accordance to the policies set by the Journal of Neuroscience Research, this manuscript presents an accurate and transparent account of the study being reported and that all critical details describing the methods and results are present.

## Author Contributions

J.L. conceptualized and wrote the manuscript, E.Z. E.O. O.G. and A.M. contributed to editing and reviewing the manuscript.

## Funding

This work was supported by NIHR Great Ormond Street Hospital Biomedical Research Centre.

## Conflicts of Interest

The authors declare no conflicts of interest.

## Supporting information


**Data S1:** Transparent Science Questionnaire for Authors.

## Data Availability

Data sharing not applicable to this article as no datasets were generated or analysed during the current study.
